# Comparison of bee products based on assays of antioxidant capacities

**DOI:** 10.1186/1472-6882-9-4

**Published:** 2009-02-26

**Authors:** Yoshimi Nakajima, Kazuhiro Tsuruma, Masamitsu Shimazawa, Satoshi Mishima, Hideaki Hara

**Affiliations:** 1 Department of Biofunctional Evaluation, Molecular Pharmacology, Gifu Pharmaceutical University, 5-6-1 Mitahora-higashi, Gifu 502-8585, Japan; 2 Nagaragawa Research Center, API Co. Ltd, 692-3 Nagara, Gifu 502-0071, Japan

## Abstract

**Background:**

Bee products (including propolis, royal jelly, and bee pollen) are popular, traditional health foods. We compared antioxidant effects among water and ethanol extracts of Brazilian green propolis (WEP or EEP), its main constituents, water-soluble royal jelly (RJ), and an ethanol extract of bee pollen.

**Methods:**

The hydrogen peroxide (H_2_O_2_)-, superoxide anion (O_2_^·-^)-, and hydroxyl radical (HO^·^)- scavenging capacities of bee products were measured using antioxidant capacity assays that employed the reactive oxygen species (ROS)-sensitive probe 5-(and-6)-chloromethyl-2',7'-dichlorodihydrofluorescein diacetate, acetyl ester (CM-H_2_DCFDA) or aminophenyl fluorescein (APF).

**Results:**

The rank order of antioxidant potencies was as follows: WEP > EEP > pollen, but neither RJ nor 10-hydroxy-2-decenoic acid (10-HDA) had any effects. Concerning the main constituents of WEP, the rank order of antioxidant effects was: caffeic acid > artepillin C > drupanin, but neither baccharin nor coumaric acid had any effects. The scavenging effects of caffeic acid were as powerful as those of trolox, but stronger than those of *N*-acetyl cysteine (NAC) or vitamin C.

**Conclusion:**

On the basis of the present assays, propolis is the most powerful antioxidant of all the bee product examined, and its effect may be partly due to the various caffeic acids it contains. Pollen, too, exhibited strong antioxidant effects.

## Background

The honeybee (*Apis mellifera*) makes various bee products from plants, flower nectar, and flower pollen, and humans make use of these products. Bee products are well known in traditional medicine, and indeed have a very long history. These days, their uses have expanded from the health food arena into the medical one. Propolis – a sticky substance that honeybees manufacture by mixing their own waxes with resinous sap obtained from the bark and leaf-buds of certain trees and other flowering plants – is used as a sealant and sterilant in honeybee nests. Now, it is recognized that propolis has a wide range of biological activities, such as antibacterial [1,2], antiinflammatory [3,4], antioxidative [5], hepatoprotective [6], and tumoricidal [7] activities.

RJ is a viscous substance secreted by the hypopharyngeal and mandibular glands of worker honeybees as an essential food for the queen bee larva and for the queen herself. RJ is composed of proteins, free amino acids, lipids, vitamins, and minerals, together with a large number of bioactive substances such as 10-HDA. RJ has several pharmacological activities, including vasodilator/hypotensive [8] and anti-tumor activities [9], and it is widely used in commercially available drugs and health foods, as well as in cosmetics, in many countries. Reported by, RJ has slight antioxidant effects, although these are weaker than those of vitamin E [10].

Bee pollen is collected by honeybees as a nutrient harvest for the hive. Pollen, the nutrients in which include proteins, amino acids, saccharides, vitamins, and minerals, is accumulated and mixed by worker honeybees with flower nectar (thus making "bee pollen") [11]. Bee pollen is considered by many to be a nutrient-rich perfect food, and is promoted as a commercially available supplement. Furthermore, Turkey bee pollen has inhibitory effects against mycelia growth of microbes and several pharmacological activities [12].

Reactive oxygen species (ROS) play key roles in many physiologic and pathogenic processes. In fact, many opthalmologic and neurodegenerative diseases seem to be mediated, at least in part, by oxidative stress [13,14]. Excess ROS generation has damage to various cell components and triggering of the activation of specific signaling pathways. Both of these effects can influence numerous cellular processes linked to ageing and the development of age-related disease [14]. H_2_O_2_, O_2_^·- ^and HO^· ^are the best-known ROS, and they can be generated either exogenously (ultraviolet light, ionizing radiation, and chemotherapeutics) or intracellularly (mitochondria, peroxisomes, lipoxygenases, NADPH oxidase, and cytochrome P450) from several different sources [14]. In recent years, Brazilian green propolis has been widely studied for its strong antioxidative effects [15-17]. However, to our knowledge, no published study has compared antioxidative effects among propolis and other bee products.

The purpose of the present study was to investigate the antioxidative effects of three representative bee products and their constituents, and to identify any ROS specifically scavenged by such bee products.

## Methods

### Materials

Drugs and sources were as follows: *p*-coumaric acid, L-ascorbic acid (vitamin C), and trolox (a water-soluble derivative of α-tocopherol) were obtained from Sigma-Aldrich (St Louis, MO, USA). *N*-Acetyl-L-cysteine (NAC) and caffeic acid were obtained from Wako (Osaka, Japan), while chlorogenic acid and quinic acid were from TC1 TOKYO KASEI (Tokyo, Japan). Artepillin C, baccharin, drupanin, 3,4-di-*O*-caffeoylquinic acid, 3,5-di-*O*-caffeoylquinic acid, and 10-HDA were gifted by API Co. Ltd. (Gifu, Japan).

### Bee products

The propolis used in the present study was Brazilian green propolis (Minas Gerais State, Brazil). This originates from *Baccharis dracunculifolia *[18], and is rich in cinnamic acid derivatives (artepillin C, baccharin, drupanin, and *p*-coumaric acid) and caffeoylquinic acid derivatives (3,4-di-*O*-caffeoylquinic acid, 3,5-di-*O*-caffeoylquinic acid, and chlorogenic acid) [18-20]. The *Baccharis *propolis was extracted either with water at 50°C (to yield WEP) or with 95% ethanol at room temperature (to yield EEP) [21]. Their main constituents were previously reported by Mishima et al. [22].

Fresh RJ, with an approximate moisture content of 67%, was obtained from *Apis mellifera *L. that had collected nectar and pollen primarily from *Brassica campestris *L (*Brassicaceae*) in the Yangtze Valley of the People's Republic of China. The RJ sample we used has been freeze-dried.

The bee pollen used in the present study originated from *Jara pringosa *(Sistus Ladanifer) and *Jara blanca *(Cistus Albidus) in Spain. It was extracted with 95% ethanol at room temperature.

### Cell culture

Retinal ganglion cells (RGC-5, a rat ganglion cell-line transformed using E1A virus) were maintained in Dulbecco's modified Eagles's medium (DMEM) containing 10% fetal bovine serum (FBS), 100 U/ml penicillin, and 100 μg/ml streptomycin. Cultures were maintained at 37°C in a humidified atmosphere of 95% air and 5% CO_2 _at 37°C, as described in our previous report [23].

### Antioxidant-capacity assay

Antioxidant-capacity assay was used to examine intracellular ROS. This assay measured the radicals induced in RGC-5 by the application of ROS (H_2_O_2_, O_2_^·-^, and HO^·^). The cells were seeded at a density of 2 × 10^3 ^cells per well into 96-well plates, and then incubated in a humidified atmosphere of 95% air and 5% CO_2 _at 37°C. Twenty-four hours later, the cell-culture medium was replaced, before any treatment with bee products or their vehicle (DMEM containing 1% FBS). After pretreatment with a bee product or its vehicle for 1 h, we added the radical probe, 5-(and-6)-chloromethyl-2', 7'-dichlorodihydrofluorescein diacetate, acetyl ester (CM-H_2_DCFDA) (Molecular Probes, Eugene, OR, USA) at 10 μM, and allowed incubation to proceed for 20 min at 37°C [24]. Then, the cell culture medium was replaced to remove the surplus probe. CM-H_2_DCFDA (inactive for ROS) is converted to DCFH (active for ROS) by being taken into the cell and acted upon by an intracellular enzyme (esterase). The H_2_O_2 _or O_2_^·- ^oxidizes intracellular DCFH (non-fluorescent) to DCF (fluorescent). To generate the ROS, we added H_2_O_2 _(Wako, Osaka, Japan) at 1 mM (H_2_O_2_) or KO_2 _(Aldrich Chemical Company, Inc., Milwaukee, Wisconsin, USA) at 1 mM (O_2_^·-^) as the radical probe loading-medium. Fluorescence was measured, after the ROS-generating compounds had been present for various time-periods, using Skan It RE for Varioskan Flash 2.4 (Thermo Fisher Scientific, Waltham, MA, USA) at excitation/emission wavelength of 485/535 nm. In addition, to detect the HO^· ^formed in the Fenton reaction, we used 2-[6-(4'-amino) phenoxy-3H-xanthen-3-on-9-yl] benzoic acid (APF) (Daiichi Pure Chemicals, Tokyo, Japan) [25]. Briefly, cells were loaded with APF by incubation for 20 min at 37°C in Hanks/Hepes buffer solution containing APF (10 μM). To perform the Fenton reaction, H_2_O_2 _was added to the Hanks/Hepes buffer solution of APF, and then iron (II) perchlorate hexahydrate (Wako) was added. Fluorescence was measured at excitation/emission wavelengths of 490/515 nm. Total intensity was calculated by integrating the area under the DCF or reactive APF fluorescence intensity curve for 20 min after treatment with ROS-generating compounds.

### Statistical analysis

Results are presented as the mean ± S.E.M. of 6 independent experiments, with each treatment performed in duplicate. Statistical significance was determined by a one-way ANOVA followed by a post-hoc Dunnett's test for comparisons of bee product versus vehicle, as indicated in the Figures (*p < 0.05, **p < 0.01).

## Results

### Effects of Brazilian green propolis on intracellular oxidation

**Figure 1:**
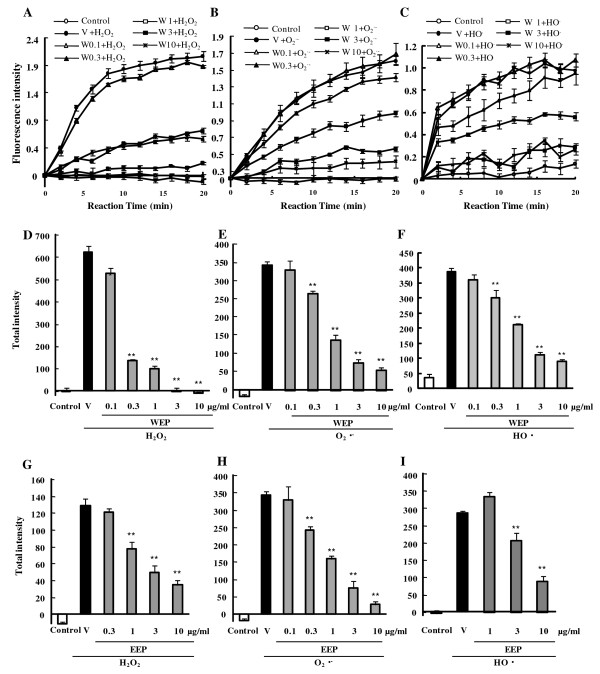
**Time-kinetic and concentration-response data for antioxidant activities of Brazilian green propolis towards production of various ROS (H_2_O_2_, O_2_^·-^, HO^·^) in term of fluorescence intensity**. (A-C) Water extract of propolis (WEP) was added to RGC-5 cultures for 1 h, followed by addition of CM-H_2_DCFDA (10 μM) or APF (10 μM) for 20 min. "Control" exhibited no ROS stimulation, while vehicle plus ROS induced ROS stimulation that was concentration-dependently reduced by WEP treatment. Kinetics of the DCFH oxidation induced by (A) H_2_O_2_, (B) O_2_^·-^, and (C) kinetics of the APF oxidation induced by HO^· ^in RGC-5. (D-F) Integrals of ROS production were calculated from the time-kinetics curves (see A-C), as described in "Methods". ROS were (D) H_2_O_2_, (E) O_2_^·-^, (F) HO^·^. (D-F) WEP. (G-I) EEP. Data are shown as mean ± S.E.M., n = 6. *P < 0.05, **P < 0.01 vs. vehicle plus ROS. V: vehicle, W: WEP, E: EEP.

To investigate the effects of WEP and EEP on the production of hydrogen peroxide (H_2_O_2_), superoxide anion (O_2_^·-^), and hydroxyl radical (HO^·^), we employed antioxidant-capacity assays using one of the two ROS-sensitive probes, CM-H_2_DCFDA or APF. The time-kinetics of ROS reactivity (monitored as fluorescence generation) are illustrated in Figure 1A–C. Pretreatment of RGC-5 with WEP at 0.1–10 μg/ml dramatically scavenged H_2_O_2 _(Fig. 1D). Similarly, pretreatment with WEP at 0.3–10 μg/ml scavenged both the O_2_^·- ^and the HO^· ^(Fig. 1E and 1F) time-dependently for 20 min. Pretreatment with EEP reduced the O_2_^·- ^somewhat more effectively than either the H_2_O_2 _or HO^· ^(Fig. 1G–I). The IC_50 _values (the concentrations causing 50% inhibition, with 95% confidence limits) for the effects of various bee products and compounds against H_2_O_2_, O_2_^·-^, and HO^· ^are given in Table 1. In its capacity to scavenge individual ROS, WEP was about the same or more effective than EEP. In particular, the H_2_O_2_-scavenging capacity of WEP indicated a toughly ten-times greater antioxidant activity than that of EEP.

**Table 1:** Antioxidant activities of bee products and antioxidants.

	IC_50 _(95% confidence limit)		
	
Compounds	H_2_O_2_	O_2_^·-^	HO^·^
Brazilian green propolis (μg/ml)			
WEP	0.24 (0.15–0.34)	0.91 (0.64–1.21)	4.12 (3.31–5.17)
EEP	2.48 (1.65–4.05)	0.79 (0.56–1.04)	5.83 (4.99–6.87)

Other bee products (μg/ml)			
Pollen	9.99 (8.01–12.3)	8.44 (6.64–10.4)	57.6 (46.5–69.9)
Royal jelly	> 100	> 100	> 100

Constituents of propolis (μM)			
3,4-di-*O*-Caffeoylquinic acid	0.52 (0.36–0.70)	0.25 (0.18–0.32)	1.86 (1.50–2.31)
3,5-di-*O*-Caffeoylquinic acid	0.33 (0.16–0.53)	0.18 (0.13–0.24)	2.02 (1.51–3.07)
3-Caffeoylquinic acid	0.22 (0.11–0.35)	0.16 (0.11–0.22)	2.38 (1.83–3.19)
Artepillin C	1.44 (1.16–1.76)	2.01 (1.38–2.84)	51.9 (39.1–73.1)
Baccharin	> 100	> 100	> 100
Coumaric acid	> 100	> 100	59.4 (39.1–102.7)
Drupanin	7.35 (5.28–10.0)	5.24 (3.20–8.00)	26.4 (17.4–43.4)
Caffeic acid	0.28 (0.22–0.36)	0.13 (0.11–0.16)	1.82 (1.50–2.23)
Quinic acid	> 100	> 100	> 100

Constituents of royal jelly (μM)			
10-Hydroxy decenoic acid	> 100	> 100	> 100

Antioxidants (μM)			
Trolox	0.29 (0.11–0.59)	0.36 (0.13–0.75)	1.30 (0.97–1.77)
NAC	1.84 (1.02–4.37)	2.40 (1.46–4.42)	> 100
Ascorbic acid (Vitamin C)	1.53 (1.38–1.70)	0.70 (0.50–0.94)	2.07 (0.98–5.81)

IC_50_: 50% inhibitory concentration, WEP: water extract of propolis, EEP: ethanol extract of propolis, NAC: *N*-acetyl cysteine,

### Effects of RJ and pollen on intracellular oxidation

**Figure 2:**
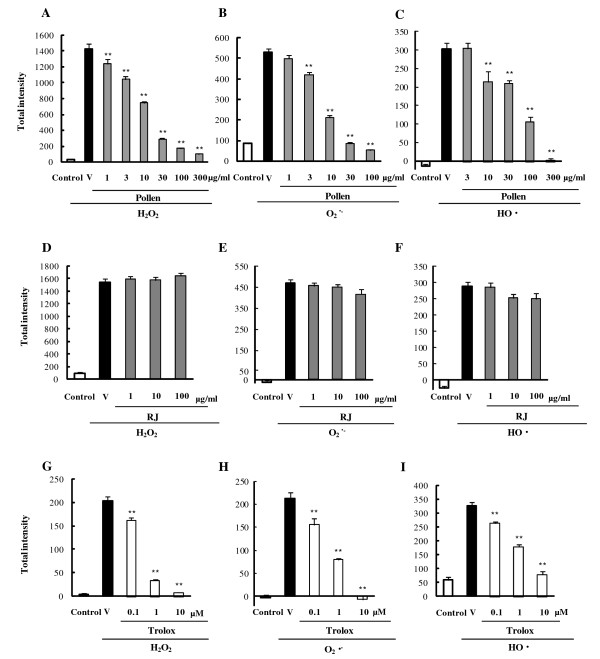
**Antioxidant activities of bee products and trolox towards production of various ROS (H_2_O_2_, O_2_^·-^, HO^·^) in term of fluorescence intensity**. (A-C) Bee pollen. (D-F) Royal jelly (RJ). (G-I) Trolox (a derivative of α-tocopherol). Integrals of ROS production were calculated from time-kinetics curves. ROS were (A, D, G) H_2_O_2_, (B, E, H) O_2_^·-^, (C, F, I) HO^·^. Data are shown as mean ± S.E.M., n = 6. **P < 0.01 vs. vehicle plus ROS. V: vehicle.

To compare the antioxidative effects of other bee products, including RJ and pollen, with those of propolis, we employed antioxidant-capacity assay. Pretreatment with pollen at 1–300 μg/ml scavenged the H_2_O_2 _in RGC-5 (Fig. 2A). Similarly, pretreatment with pollen at 3–100 μg/ml reduced the O_2_^·- ^(Fig. 2B), while pollen at 10–300 μg/ml reduced the HO^· ^(Fig. 2C). RJ with IC_50 _values of more than 100 μg/ml, did not scavenge any of the ROS (Fig. 2D–F). From the IC_50 _values given in Table 1, there are marked differences in antioxidant activities among bee products, the rank order being: propolis > pollen > RJ. Notably, propolis and pollen each exhibited weaker scavenging activity against the HO^· ^than against H_2_O_2 _and O_2_^·-^.

### Effects of main constituents of WEP (caffeoylquinic acid derivatives) on intracellular oxidation

**Figure 3:**
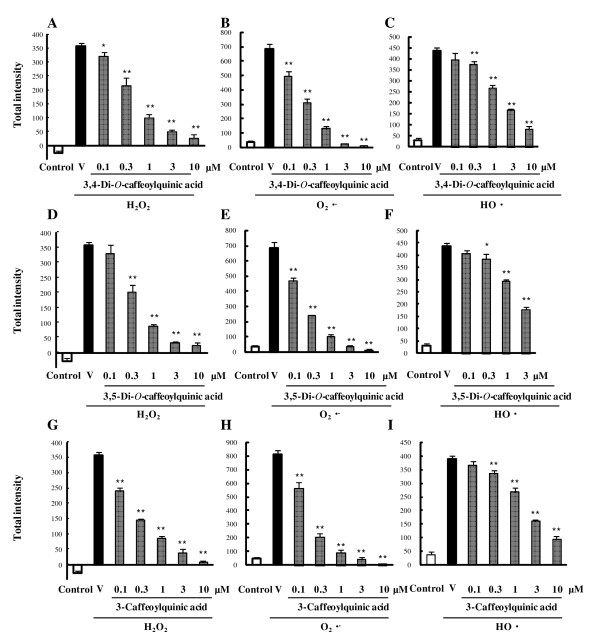
**Antioxidant activities of main constituents of WEP (caffeoylquinic acid derivatives) towards production of various ROS (H_2_O_2_, O_2_^·-^, HO^·^) in term of fluorescence intensity**. (A-C) 3,4-di-*O*-caffeoylquinic acid. (D-F) 3,5-di-*O*-caffeoylquinic acid. (G-I) 3-caffeoylqic acid (Chlorogenic acid). Integrals of ROS production were calculated from time-kinetics curves. ROS were (A, D, G) H_2_O_2_, (B, E, H) O_2_^·-^, (C, F, I) HO^·^. Data are shown as mean ± S.E.M., n = 6. *P < 0.05, **P < 0.01 vs. vehicle plus ROS. V: vehicle.

To investigate which WEP constituents might be responsible for its strong antioxidative effects, we examined the antioxidant effects of three main constituents of WEP (3,4-di-*O*-caffeoylquinic acid, 3,5-di-*O*-caffeoylquinic acid, and 3-caffeoylquinic acid) (Figure 3). Each caffeoylquinic acid derivative significantly reduced all three ROS (H_2_O_2_, O_2_^·-^, and HO^·^). Specifically, H_2_O_2 _and O_2_^·- ^were strongly scavenged by mono-caffeoylquinic acid (3-caffeoylquinic acid), while the HO^· ^was strongly scavenged by the two di-caffeoylquinic acids (3,4- and 3,5-di-*O*-caffeoylquinic acid). All three caffeoylquinic acid derivatives scavenged the O_2_^·- ^more effectively than the other ROS (Table 1). These results indicate that the potent antioxidative activities of WEP may be due to those of the caffeoylquinic acid derivatives present in WEP.

### Effects of main constituents of EEP (cinnamic acid derivatives) on intracellular oxidation

To investigate which EEP constituents might be responsible for its strong antioxidative effects, we examined the antioxidant effects of the main constituents of EEP (artepillin C, baccharin, *p*-coumaric acid, and drupanin). Pretreatment with artepillin C at 0.1–100 μM scavenged the H_2_O_2 _and O_2_^·-^, while at 10–100 μM it scavenged the HO^·^. Thus, artepillin C exerted strong antioxidant effects, especially against the H_2_O_2 _and O_2_^·-^. Pretreatment with drupanin at 1–100 μM concentration-dependently scavenged each ROS (H_2_O_2_, O_2_^·- ^and HO^·^). Pretreatment with *p*-coumaric acid or baccharin had little or no effects on the three ROS. The results show clear differences in antioxidant activities among cinnamic acid derivatives (main constituents of EEP), the rank order being: artepillin C > drupanin > *p*-coumaric acid and baccharin (Table 1). These results indicate that the potent antioxidative effects of EEP may be partly due to those of artepillin C and drupanin.

### Effects of metabolites of caffeoylquinic acids (caffeic and quinic acids) on intracellular oxidation

Caffeoylquinic acid derivatives consist of caffeic and quinic acids. Caffeic acid which is a cinnamic acid derivative has a strong antioxidant and antioxidant properties [26]. To examine whether metabolic derivatives of caffeoylquinic acid are responsible for its antioxidative effects, we evaluated the antioxidant effects of caffeic and quinic acids. The antioxidative effects of caffeic acid were either equal to or greater than those of the caffeoylquinic acids or their other derivatives (Table 1). Quinic acid, with IC_50 _values of more than 100 μM, did not scavenge any of the ROS.

### Effects of representative antioxidants on intracellular oxidation

As a step in the standardization of the antioxidative activities of bee products (including propolis, RJ, pollen, and the major constituents of propolis), we examined the effects of the representative free-radical scavengers, trolox, NAC, and vitamin C. Pretreatment either with trolox at 0.1–10 μM (Fig. 2G–I) or with vitamin C at 1–10 μM (Table 1) trapped the H_2_O_2_, O_2_^·-^, and HO^·^, while NAC at 0.1–10 μM scavenged only the H_2_O_2 _and O_2_^·- ^(Table 1). These results reveal antioxidant activities with the following rank order: trolox > vitamin C > NAC. Our data also indicate that caffeic acid and caffeoylquinic acid derivatives had antioxidative effects as strong as those of trolox. The antioxidative effects of artepillin C and drupanin were about the same as, or more effective than, those of vitamin C and NAC. Thus, the above constituents of propolis are about as potent as antioxidants as the typical antioxidants tested here.

## Discussion

Oxidative stress, which may be defined as an imbalance between the production and removal of ROS, has been implicated in many types of nerve-cell death, both within the central nervous system and in the eye [27]. A previous report examined a variety of ROS-generating mechanisms for their involvement in retinal ischemia, and the effects of neuroprotective agents against such damage were also examined [28]. In previous report, antioxidative effects of EEP are measured using chemiluminescence assay. Pretreatment with EEP scavenged the all ROS, although the IC_50 _values are different from our results. These assay may probably be due to the pH of the medium which permitted different redox potential of the propolis antioxidant compounds, and also due to the different kind of radicals formed [29]. Our results may be more reflected in biochemical reactions within the body because our study was measured using living cells. We therefore used RGC-5 to investigate effects on endogenously generated ROS, since we felt that such an examination was likely to help clarify the effects of bee products and their constituents on disease processes.

Our results demonstrate that propolis (both WEP and EEP) had the strongest antioxidant effects (against H_2_O_2_, O_2_^·-^, and HO^·^) among the bee products tested (propolis, RJ, and bee pollen). Bee pollen had fairly strong antioxidant effects, especially against the H_2_O_2 _and O_2_^·-^, although its effects were only one-tenth as powerful as those of propolis. On the basis of their IC_50 _values (> RJ at100 μg/ml and > 100 μM, respectively), RJ and its main constituent, 10-HDA, did not scavenge any ROS. It has been reported that in tissue DNA-damaged mice, dietary RJ reduced the 8-hydroxy-2-deoxyguanosine (8-OHdG) levels in both kidney DNA and serum [30]. Although RJ displayed very little potency at scavenging any ROS in our experiment, it is possible that, dietary RJ exerts protective effects against tissue damage in the body through other mechanisms other than ROS scavenging.

Recently, we reported that both WEP and EEP displayed antioxidant actions against lipid peroxidation in mouse forebrain homogenates and against the diphenyl-*p*-picrylhydrazyl (DPPH) radical [16]. Those antioxidant activities of WEP and EEP were almost as powerful as the ones in this report. Collectively, therefore our data indicate that WEP and EEP have potent antioxidant effects against a variety of ROS. In the present study, we also examined the antioxidant effects of certain propolis constituents in detail, to clarify the factor(s) contributing to the antioxidant effects of propolis itself. The main constituents of WEP [caffeoylquinic acid derivatives (both mono-caffeoylquinic acid and di-caffeoylquinic acids)] were found to have antioxidant effects with efficacies about the same as those of trolox. These constituents may be primarily responsible for the powerful antioxidative effects of WEP (considering that they are at high percentage levels as constituents of WEP) [21].

Caffeoylquinic acid derivatives are metabolized to caffeic and quinic acids in human serum [31]. In the present study, quinic acid (IC_50 _> 100 μM) did not scavenge any of the ROS, whereas caffeic acid dramatically reduced all three ROS. These results indicate that the strong antioxidative effects of caffeoylquinic acid derivatives within the human body may be due to the hydroquinone moiety of caffeic acid. Interestingly, our results showed that di-caffeoylquinic acid, despite including two caffeic acids, had weaker antioxidant effects than either mono-caffeoylquinic acid or caffeic acid. Possibly, these differences may be causally related to conformational interference.

In the present study, the scavenging effects of caffeic acid were of equivalent efficacy to those of trolox. It has been reported that caffeic acid increases the expression of glucose-6-phosphate dehydrogenase (G6PD), known to be an antioxidant gene that is stronger than trolox [32]. In antioxidant-capacity assay using stable green radical cation of 2',2'-azinobis-(3-ethylbenzothiazoline-6-sulphonic acid) (ABTS), caffeic acid and chlorogenic acid are stronger than ascorbic acid [33]. Similarly, our results indicated that antioxidative effects of caffeic acid and chlorogenic acid against H_2_O_2 _and O_2_^·- ^were 4–6 times stronger than ascorbic acid (Table 1). Therefore, caffeic acid may have greater beneficial antioxidant effects than many other antioxidants.

Artepillin C, a main constituent of EEP, was found to have strong antioxidant effects, but neither baccharin nor *p*-coumaric acid had such effects. Therefore, artepillin C may be partly responsible for the potent antioxidant effects of EEP. Reportedly, EEP contains caffeoylquinic acid derivatives at one-half the amounts found in WEP [21]. Although artepillin C had only a slight HO^· ^antioxidant effect, EEP had a very potent antioxidative effect against the HO^·^. We had considered that the caffeoylquinic acid derivatives contained by EEP were effectively responsible for its HO^· ^antioxidant activity. On the basis of the above data, we now assume that the strong antioxidant effects of EEP may be accounted for by additive effects of caffeoylquinic acid and prenyl analogues, including artepillin C.

## Conclusion

We found that among the bee products tested, propolis had the strongest antioxidant effects. Caffeoylquinic acid derivatives, main constituents of propolis, have strong antioxidative effects and equivalent efficacy of trolox and ascorbic acid. Furthermore, since propolis and its constituents were widely effective as an antioxidant [i.e., it scavenged all three ROS (H_2_O_2_, O_2_^·-^, and HO^·^)] it may be expected to have beneficial effects against at least some oxidative stress-related diseases.

## Competing interests

The authors declare that they have no competing interests.

## Authors' contributions

YN performed the study and wrote the paper; KT, MS and SM participated in the design of the study, and acquisition of sample; HH conceived of the study, and participated in its design and coordination. All authors read and approved the final manuscript.

## Pre-publication history

The pre-publication history for this paper can be accessed here:


